# Degenerative Aortic Stenosis, Dyslipidemia and Possibilities of Medical Treatment

**DOI:** 10.3390/medicina54020024

**Published:** 2018-04-25

**Authors:** Rita Kleinauskienė, Regina Jonkaitienė

**Affiliations:** Department of Cardiology, Medical Academy, Hospital of Lithuanian University of Health Sciences, Eivenių g. 2, 50161 Kaunas, Lithuania; jonkaitiene.regina@gmail.com

**Keywords:** degenerative aortic stenosis, lipoproteins, dyslipidemia, statins, medical treatment

## Abstract

Degenerative aortic stenosis (DAS) is the most frequently diagnosed heart valve disease in Europe and North America. DAS is a chronic progressive disease which resembles development of atherosclerosis. Endothelial dysfunction, lipid infiltration, calcification and ossification are evidenced in both diseases. The same risk factors such as older age, male sex, smoking, and elevated levels of lipids are identified. The effect of smoking, visceral obesity, metabolic syndrome, hypercholesterolemia, low-density lipoprotein, high-density lipoprotein, lipoprotein(a), adiponectin and apolipoprotein(a) on development of DAS are being studied. The search for genetic ties between disorders of lipid metabolism and DAS has been started. DAS is characterized by a long symptom-free period which can last for several decades. Aortic valve replacement surgery is necessary when the symptoms occur. The lipid-lowering therapy effect on stopping or at least slowing down the progression of DAS was studied. However, the results of the conducted clinical trials are controversial. In addition, calcium homeostasis, bone metabolism and calcinosis-reducing medication are being studied. Although prospective randomized clinical trials have not demonstrated any positive effect of statins used for slowing progression of the disease, statins are still recommended for patients with dyslipidemia. Recent study has suggested that a specific modification of treatment, based on severity of disease, may have a beneficial effect in patients with aortic sclerosis and mild DAS. New clinical studies analyzing new treatment possibilities which could correct the natural course of the disease and reduce the need for aortic valve replacement by surgery or transcatheter treatment interventions are needed.

## 1. Introduction

Degenerative aortic stenosis (DAS) is the third cause of death among cardiovascular diseases, and morbidity with DAS has been rapidly increasing for the last decade [1,2]. DAS represents the narrowing of the aortic valve, which causes an obstruction of the left ventricular outflow and eventually developing symptoms of the disease [2]. It is the most frequently diagnosed aortic valve disease in Europe and North America. Prevalence of DAS is 2–7% [3] or, according to other data, 8% of adults who are over 65 years old [1]. According to the data of 2017, moderate and severe DAS prevalence was 2.8% of adults over 75 years old [4]. Prevalence of DAS was almost 10% of adults who are over 80 years old [5]. It was determined in a prospective Cardiovascular Health Research study (CHR) that aortic sclerosis prevalence was 26% and DAS–2% of 5,201 patients who were over 65 years old. Aortic sclerosis prevalence was 48% and DAS–4% of the patients who were over 85 years old [6]. DAS is characterized by a long symptom-free period which can last for several decades [2]. Then symptoms occur and urgent intervention is necessary [2,5]. Symptomatic DAS mortality is almost 50% in two years unless valvular stenosis is relieved by intervention (aortic valve replacement or transcatheter aortic valve implantation) [2,5]. Meanwhile, asymptomatic patients must be constantly monitored for progression of DAS and occurrence of clinical symptoms [1].

Rheumatic aortic valve stenosis was predominant in developed countries until 1970. Currently, the predominant cause of aortic valve stenosis is degeneration and calcinosis of aortic valve [6,7]. Historically, DAS has been considered a consequence of long-term “wear” and age-related degeneration of valves [6,7,8,9]. Calcinosis of aortic valve was the disease of the elderly; however, it has been proven that DAS is not a consequence of aging only [7]. Histopathological tests have shown that calcinosis of aortic valve is an active process which resembles development of atherosclerosis [10,11]. Endothelial dysfunction, lipid infiltration, calcification and ossification are evidenced in both diseases [10,12]. It has been noticed that coronary artery disease (CAD) and atherosclerotic changes in aorta are often diagnosed meanwhile for patients with DAS. It has been determined that dyslipidemia was more frequently diagnosed for the patients with an aortic valve prosthesis with or without coronary artery bypass grafting (CABG) surgery than for those for whom CABG only was performed. Therefore, it is believed that DAS and CAD share common etiological factors [5,6,10,12]. In the CHR study, it was determined that DAS is significantly related to older age, male sex, smoking, arterial hypertension (AH) as well as elevated levels of lipoprotein(a) (Lp(a)) and low-density lipoprotein (LDL). Special focus is given to the influence of cholesterol [6,10]. Lipid theory in the development of DAS is analyzed. The possibility to stop or at least slow down progression of DAS with statins was investigated; however, the results of the conducted clinical studies are controversial [10].

## 2. Degenerative Aortic Stenosis

Normal AVA in adults is 3.0–4.0 cm [1]. DAS is a chronic progressive disease [1,13]. It has been determined that the aortic valve area (AVA) narrows down by 0.05 cm^2^, whereas the velocity of blood flow through AV increased by 0.22 m/s per year on the average [14]. AV degeneration starts as AV sclerosis. Coffey et al. conducted a systematic review and meta-analysis and determined that aortic sclerosis in 1.8–1.9% of patients progresses to DAS within one year [15]. It has been determined that aortic sclerosis in one sixth of patients progresses more rapidly and AV calcinosis develops [8,16]. As DAS progresses, valve leaflets thicken and fibrose as well as calcium foci and new blood vessels are formed [6,8,17]. Calcium is accumulated on the side of aortic valve and stretches up to the sinuses of Valsalva [6,17]; therefore, movement of valve leaflets is restricted, effective aortic valve area is reduced and obstruction develops in the left ventricle (LV) outflow tract [6,8,18].

DAS is asymptomatic for a long period. This latent period is individual for each patient [1]. Mortality of asymptomatic patients is similar to that of patients of respective age. Risk of sudden death is low (<1% per year). Degree of aortic valve calcinosis and severity of DAS directly correlate with clinical manifestation [1,3]. There are three typical clinical symptoms of DAS: dyspnea, angina pectoris and syncope. Dyspnea and, therefore, reduced physical activity is developing first, whereas syncope, angina pectoris are developing later [1,6]. However, if surgical or transcatheter treatment is not applied after symptoms develop, progress of the disease worsens significantly [3]. Five–year survival rate of patients with flow velocity through AV less than 3 m/s is 75–85%, whereas survival of patients with flow velocity through AV more than 4 m/s is 30–50% only [1,3].

In DAS, the functional AVA is decreased sufficiently to cause measurable obstruction to outflow and significant gradient from left ventricle to aorta [16]. DAS is one of the most common causes of LV pressure overload [19]; therefore, LV hypertrophy (LVH) occurs [1,6]. LVH is compensative and helps maintain stable systolic blood volume of the heart and sufficient LV systolic function [19]. LVH covers not only myocytes but also non-muscular components of LV, composition of intercellular space, rigidity of LV increases, fibrosis develops, relaxation of LV decreases and diastolic function fails. As the disease progresses, hypertrophied LV walls are not able to maintain sufficient intraventricular systolic pressure, systolic function of LV fails and heart failure develops [6]. Systolic and diastolic dysfunction significantly worsens survival prognosis [19].

## 3. Pathogenesis of Degenerative Aortic Stenosis

Scientific research has proven that development of DAS is similar to the development process of atherosclerosis [2,7,11,18]. It has been determined that both AV calcinosis and atherosclerosis cover endothelial dysfunction, lipid infiltration, inflammation, neoangiogenesis and calcinosis. CAD and DAS are frequently evidenced together [2,7,12,20]. It is known that intraplaque haemorrhage plays an important role in the progression of atherosclerosis. Recent study clarified that intraleaflet haemorhage was frequently observed in the valve leaflets of DAS and associated with a rapid progression of DAS [21]. Besides, study demonstrated that valvular interstitial cells (VIC) can take up and accumulate iron, which resulted in increased proliferation and decreased elastin production, so iron transport can have a major impact on DAS [22]. However, there are some significant differences between atherosclerosis of blood vessels and degeneration of aortic valve. In the case of CAD, clinically significant events are caused by rupture of atherosclerotic plaque, whereas, in the case of DAS, progressing valve calcinosis reduces mobility of valve leaflets and disturbs their function. CAD and DAS share the same pathophysiological grounds; however, their development and progression mechanisms are different at tissue level [2,7,17]. DAS development is initiated by endothelial dysfunction, inflammatory process and lipid infiltration [7], whereas progression is induced by mechanical stress, genetic factors as well as interaction between inflammatory and calcinosis processes [7,18].

DAS is initiated in the vascular side of the leaflets with focal subendothelial lesions that are similar to atherosclerosis plaques of CAD [6,7,17]. The initial aortic lesions contain disorganized collagen fibers, chronic inflammatory cells, lipids and proteins of extracellular matrix and bone minerals [6,7]. It is known that DAS develops earlier for those patients who have congenital bicuspid aortic valve due to higher share stress on valve leaflets [6,7,17,18].

**Figure 1:**
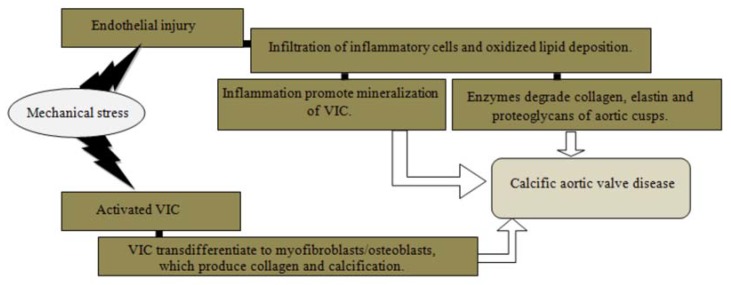
Pathogenesis of calcific aortic valve disease.

The most common cause of aortic valve stenosis is valve calcification, termed calcific aortic valve disease (CAVD) (Figure 1). CAVD is an active cellular biological process characterized by alterations of the cells of aortic valve [6,17,20]. Mechanical stress activates VIC, induces proliferation and mineralization [7] as well activates myofibroblasts and osteoblasts, promoting calcification, osteogenesis and bone formation [6,16,17]. In two clinical studies analyzing 1524 stenotic aortic valves, formed bone tissue was determined in 10.9–13% of cases [6].

Another development mechanism is based on endothelial damage due to mechanical stress [6,7,8,16]. Hemodynamic damage leads to activated lipid (LDL and Lp(a)) infiltration and which undergo oxidative modifications. These oxidized lipoproteins are highly cytotoxic and are able to stimulate both the inflammatory response and the mineralization activity [2,7,8,16,18,20]. Once inflammatory cells, like macrophages, T lymphocytes, monocytes, are recruited in the endothelium, they release enzymes, such as matrix metalloproteinase, that degrade collagen, elastic fibers and proteoglycans of the aortic valve leaflets [2,6,16,20,23]. Released cytokines stimulate development of aortic valve fibrosis and calcinosis, so leaflets become thickened, fibrosed, and calcified, resulting in reduced leaflet mobility and progressive valvular obstruction [7,8,16,17,20].

## 4. Lipid Theory

Various theories concerning the occurrence and progression of calcinosis and degeneration of aortic valve are being studied widely (Table 1). The effect of alcohol consumption, smoking, visceral obesity, metabolic syndrome (MS) on development of DAS as well as lipid theory assessing the relation between DAS and not only hypercholesterolemia, LDL, high-density lipoprotein (HDL) and triglycerides (TG), but also Lp(a), adiponectin and apolipoprotein(a) are being studied. The study for genetic ties between disorders of lipid metabolism and DAS has been started.

In the study with mice it was determined that visceral obesity, MS and high fat/high carbohydrate diet induce development of DAS [24]. Larsson et al. studied the association of overall and abdominal obesity with DAS and has been proven that obesity is associated with increased risk of DAS [25]. Recent study was investigated the associations of smoking with risk of DAS. It was determined that risk of DAS increased with increasing smoking intensity and former smokers had a similar risk of developing DAS as a newer smoker [26]. It was determined that DAS progresses more rapidly in case of MS than for those without MS irrespective of LDL levels. In case of MS, flow velocity through AV increases by 0.25 ± 0.21 m/s per year on average, while for those without MS–0.19 ± 0.19 m/s per year (*p* = 0.03) [27]. Even in cases when intensive lipid-reducing therapy is applicated and recommended LDL levels are achieved, DAS progresses twice as fast as for patients without MS [11].

Many studies proving the effect of lipid metabolism disorders have been conducted. In 1997, it was determined in vitro that oxidized lipids stimulate calcinosis [28], whereas a study conducted on animals has proved that hypercholesterolemia induced calcinosis and DAS [29]. Lipids have a significant role in pathogenesis of DAS via molecular processes of cells, e.g., Wnt/Lrp5 and RANK/RANKL/osteoprotegerin which induce transition of valvular myofibroblasts to osteogenic components due to which accumulation of bone tissue begins in valves [17,30].

It was determined in CHR and MONIKA/KORA studies that cholesterol and related lipoproteins are independent DAS risk factors [31,32]. Parisi et al. analyzed the interaction of lipids, inflammatory process and DAS. It was proven that increased LDL levels induce development of not only CAD, but also DAS. It was determined that lipids activate biological molecular processes as well as activate calcinosis of aortic valve. Lipid metabolism disorders influence calcinosis of blood vessels and valves as well as altered metabolism of bone proteins [30]. 

The fact that hypercholesterolemia influences not only CAD but also calcinosis of the root of aorta and, especially, aortic valve has been proven for patients suffering from homozygous familial hypercholesterolemia. Lp(a) concentration is an independent risk factor for development of aortic valve calcification, DAS and unfavorable prognosis [7]. In EPIC-Norfolk and two prospective general population studies (the Copenhagen City Heart Study (CCHS) (1991–2011; *n* = 10,803) and Copenhagen General Population Study (CGPS) (2003–2011; *n* = 66,877) determined that elevated Lp(a) levels increase the risk of valve calcinosis [33,34]. An analysis of 129 Dutch people suffering from familial hypercholesterolemia was conducted recently and it was determined that elevation of Lp(a) by 10 mg/dL (0,259 mmol/L) is associated with 11% likelihood of DAS development [29]. MESA study has proven a significant correlation between Lp(a), the degree of calcinosis of aortic valve and progression of DAS irrespective of patients’ race, age, sex, arterial hypertension, diabetes, smoking, HDL and TG [35]. Previous study also pointed out genetic ties between Lp(a) and DAS [36]. CHARGE study determined that genetic variants of Lp(a) are strongly related to calcinosis of aortic valve and DAS [37]. The study has proven that increase of Lp(a) directly induces calcinosis of aortic valve and progression of DAS [38]. The results of the study promoted assessment of Lp(a) as a factor which can modify AV disease. Thanassoulis et al. argue that targeted Lp(a) therapy may become a new opportunity to treat DAS [39]. Penetration and oxidation are significant pathogenic mechanisms which stimulate inflammatory reaction in aortic valve endotelium. Close relationship between lipids, inflammation and DAS was determined [7,11,12]. LDL and Lp(a) infiltrate the location of injury and oxidize. These oxidized lipoproteins are cytotoxic and they stimulate inflammatory response and mineralization [2,7,12,30].

Genetic relationship between LDL and DAS has been proven by other studies. Smith et al. have determined a genetic causal relationship between elevated LDL concentration and calcinosis of aortic valve as well as DAS. It was determined that LDL, but not HDL or TG, is significantly related to DAS (HR 1.28; 95% CI, 1.04–1.57; *p* = 0.02). Genetic risk score (GRS) of LDL (but not that of HDL or TG) was significantly associated with calcinosis of aortic valve in CHARGE study (OR 1.38; 95% CI, 1.09–1.74; *p* = 0.007) as well as with DAS in MDCS study (HR 2.78; 95% CI, 1.22–6.37; *p* = 0.02). GRS of LDL was significantly related to calcinosis of aortic valve (*p* = 0.03) and AS (*p* = 0.009) [37]. Recent study confirmed the association between 2 LPA variants (rs10455872 and rs 3798220) and aortic stenosis. In this study it has been proven that individuals with two risk alleles have 2-fold greater odds of developing aortic stenosis [40].

The search for the factors which stimulate calcinosis of aortic valve and progression of the disease is still ongoing. Dyslipidemia has been proven to significantly stimulate progression of DAS. Kamath et al. have determined that DAS progresses twice as rapidly in cases of hypercholesterolemia [41]. When cholesterol levels exceed the threshold of 200 mg/dL (5.18 mmol/L), AS progresses twice as rapidly than in patients without hypercholesterolemia [2]. Calcinosis of aortic valve progressed by 9 ± 2% per year for patients with lower LDL level (<130 mg/dL or <3.37 mmol/L) on the average, whereas calcinosis of aortic valve progressed by 43 ± 44% per year for patients with higher LDL level (>130 mg/dL or >3.37 mmol/L) on the average (*p* < 0.001) [7]. Kücük et al. assessed the influence of LDL, HDL and total cholesterol/DTL ratio on progression of DAS taking into account hemodynamic indicators. Alteration of mean pressure gradient through aortic valve (mpg) per year is mostly related to HDL concentration (r = −0.528; *p* = 0.001) and total cholesterol/HDL ratio (r = +0.505; *p* = 0.001). Weak positive correlation with LDL concentration was determined (r = 0.325; *p* = 0.036). Maximum progression of flow velocity through AV (Vav max) is mostly related to LDL (r = 0.328; *p* = 0.034), total cholesterol/HDL ratio (r = 0.499; *p* = 0.001), whereas weak negative correlation with HDL concentration was determined (r = −0.464; *p* = 0.002) [42].

Besides the factors which induce calcinosis of aortic valve and progression of DAS, the search for the factors which would stop DAS is also ongoing. There is not enough evidence yet; however, it is highly probably that HDL is one of the most significant factors causing the relationship between MS and DAS. HDL is characterized by anti-inflammatory and anti-atherogenic effect [27,42]. Studies in vitro have determined that HDL is characterized by anti-calcinotic properties. It protects LDL from the oxidation process [42]. Besides, recent studies have emphasized the effect of apolipoprotein(a) as an anti-inflammatory component of HDL which does not depend on HDL cholesterol level [11,27]. It has also been determined that HDL regulates transformation of the cells of blood vessels to osteoblastic ones; therefore, the concept that the field of application of HDL is broader than merely return transportation of cholesterol is supported; hence, further research in search for new treatment opportunities are needed [11,42].

Another factor characterized by anti-sclerotic, anti-diabetic features is adiponectin. Adiponectin is a protein produced by adipocytes and which circulates in plasma. Visceral obesity is related to reduced adiponectin levels. It has been determined that low adiponectin level is a significant and independent factor which induces progression of DAS. It has also been proven that low adiponectin level is related to increased activity of inflammatory process in aortic valve tissue. Therefore, it is hypothesized that adiponectin may protect aortic valve from inflammation and calcinosis [11].

A large number of completed and ongoing studies assessing the role of lipoproteins in development and progression of DAS helps to understand pathogenesis of this valvular heart disease better and motivates the search for new treatment opportunities and the need to conduct new clinical research [36].

## 5. Possibilities and Prospects of Medical Treatment

Aortic valve replacement intervention is still the only effective treatment method, and the gold standard for symptomatic DAS [3,4,6,36,43]. DAS is one of the most common causes of AV prosthesis implantation in western countries [8]. Implantation of AV prosthesis extends survival up to eight years on average [15,43]. If no surgical or interventional treatment is applied, survival of patients suffering from severe symptomatic DAS is 1–4 years [43]. Medical treatment of severe symptomatic DAS is ineffective due to mechanical obstruction of the LV outflow tract [7,11]. Treatment focuses on reducing cardiovascular risk factors, including hypertension, diabetes, smoking, dyslipidemia, obesity and low physical activity [6,30].

In asymptomatic DAS, there is no effective medical therapy which would stop progression of DAS [6]. Study on animals has determined that lipid-reducing therapy slowed down progression of the disease, but it did not have any effect on regression of the disease in mice which had hypercholesterolemia [13] (Table 2). Therefore, anti-inflammatory and lipid-reducing effect of statins was expected to be effective not only for treatment of dyslipidemia but also in slowing down the progression of DAS [7,10]. This concept was based on experimental and four retrospective clinical studies which determined slower progression of hemodynamic parameters for patients with DAS treated with statins [10,17]. A prospective non-randomized study RAAVE also determined that in patients with hypercholesterolemia who were treated with rosuvastatin, DAS had slower progression than in those without hypercholesterolemia and who had not been treated with rosuvastatin. Presumably, statin therapy is useful in the case of hyperlipidemia only [44]. However, randomized prospective clinical trials SALTIRE and SEAS have not found any effect in application of intensive lipid-reducing therapy in the case of DAS. Atorvastatin 80 mg was given during the first study, whereas simvastatin/ezetimibe 40/10 mg per day was given for treatment of DAS during the second study [45,46]. Comparison of the protocols of the studies showed that patients with hyperlipidemia were not included in these randomized prospective studies [2,10]. Similar results were obtained in the randomized prospective clinical study ASTRONOMER [47]. A systematic overview of the studies investigating the effect of statins in DAS showed that positive effect of statins was obtained in retrospective non-randomized studies only and it was not confirmed by prospective randomized studies [48].

It is not yet clear whether statin therapy can stop the progression of DAS. In fact, although the first studies are not in favor of the use of statins, some scientists are certain that statin therapy can have a positive effect if statins are prescribed in the early stage of DAS when inflammation prevails and the calcinosis process has not started yet [7,30,37]. Recent study was determined that the lipid-lowering therapy effect on slowing DAS progression increased with higher pretreatment LDL and lower peak aortic jet velocity. It has been proven that treatment stopped progression of mild DAS in the highest quartile of LDL (0.06 m/s per year slower progression than placebo in peak aortic jet velocity, *p* = 0.03), but not in the three other quartiles of LDL. There was no detectable effect of treatment among patient with moderate AS [49]. Pawade et al. also suggest statins and Lp(a) inhibitors for treatment of DAS in the first stage [18].

Although lipids, especially oxidized ones, initiate development of DAS, a positive effect is not obtained with statins; therefore, current attempts are being made to discover other causes of calcinosis and predisposing factors which induce accumulation of calcium in the tissue of aortic valve as well as the treatment measures which could stop progression of the disease and perhaps even cause regression of DAS. Calcium homeostasis and bone metabolism are being researched [7]. Researchers propose calcinosis-reducing medications (bisphosphonate, denosumab, ectonucleotidase, and ACE (angiotensin converting enzyme) inhibitors) for treatment of DAS in the calcinosis stage [7,18]. Studies concluded that vitamin D receptor genotype predisposes to the development of calcific aortic valve stenosis and B allele of the vitamin D receptor is more common in patients with DAS [18]. Synetos et al. evaluated the anticalcific action of bisphophonates on the aortic valve in an experimental model of aortic stenosis and found that biphosponates on the aortic valve can inhibit calcification in an experimental model of aortic stenosis [50]. Several small retrospective studies of the aimed at assessment of bisphosphonate stopping effect on progression of DAS have already been conducted. The results obtained from MESA recently showed that the use of bisphosphonates can slow down calcinosis of aortic valve for women over 65 years old. Bisphosphonates have a similar effect to that of statins; they affect lipid metabolism and inflammation. Bisphosphonates also suppress bone resorption and slow down release of calcium phosphates from bone tissue which may play an important role in slowing down sedimentation of bone tissue in non-bone (blood vessels or heart valves) tissues [7].

In addition, studies showed that the renin-angiotensin system (RAS) is also implicated in DAS pathogenesis. Angiotensin converting enzyme (ACE) is expressed and colocalized with LDL in calcified aortic valves [16,23]. Cote et al. conducted a study and confirmed that the use of angiotensin receptor blockers was associated with lower fibrosis score of the aortic valve inflammation and expression of interleukin-6. The study suggested that medication may alter the fibrotic process, by lowering tissue inflammation, of the aortic valve calcification [51]. An observational study showed slowing of progression of DAS in patients taking RAS inhibition by angiotensin converting enzyme inhibitors compared to those not on this therapy [16]. Andersson et al. conducted the systematic review and meta-analysis about RAS inhibitors safety and the prognostic benefit, and confirmed that use of RAS inhibitors may be safe in patients with DAS and may reduce the need for AVR, but evidence is overall weak [52].

Statins are not used if there are no other indications, in the case of DAS [2,7]. Based on the recent guidelines for the management of valvular heart disease, no medical therapy for aortic stenosis can improve outcome compared with natural history [1,53]. However, in patients who already receive a statin, because of underlying dyslipidemia or coronary artery disease a continuation of this therapy should be considered [1,3,54].

At least three more clinical studies analyzing the effect of statins on progression of DAS (STOP–AS, AORTICA, STAAT) are currently being conducted [2,55]. Two new clinical studies analyzing the effect of Lp(a)-reducing therapy on progression of DAS have also been initiated [56,57]. A study analyzing the effect of anti-osteoporosis medications on progression of DAS is also being conducted [53].

## 6. Conclusions

Although prospective randomized clinical trials have not demonstrated any positive effect of statins as regards stopping progression of DAS and no medical treatment is recommended for aortic stenosis by recent guidelines, the hypothesis that a positive effect may be achieved if statins are prescribed in the beginning of aortic sclerosis or mild DAS, when the predominant process is inflammation not calcinosis, still persists. In addition, recent study on lipid-lowering therapies modifications suggested that a specific modification of treatment based on severity of disease may have a beneficial effect in patients with aortic sclerosis and mild aortic stenosis. Finally, new clinical studies analyzing new treatment possibilities which could correct the natural course of DAS and reduce the demand for aortic valve replacement interventions are needed in order to stop the process of aortic valve calcinosis.

## Figures and Tables

**Table 1:** Studies assessing the role of various factors in development and progression of DAS.

Study	Date	Results
Kolasa-Trela et al.	2011	Low adiponectin level is a significant and independent factor which induces progression of AS [11].
Weiss et al.	2013	Wnt/Lrp5 and RANK/RANKL/Osteoprotegerin induce myofibroblasts modification to osteogenic components [17].
Drolet et al.	2006	VS, MS and high fat/high carbohydrate diet induce development of DAS [24].
Larsson et al.	2017	Obesity is associated with increased risk of DAS [25].
Larsson et al.	2017	Risk of DAS increased with increasing smoking intensity and former smokers (who had quit smoking 10 or more years ago) had similar risk for DAS as newer smoker [26].
Capoulade et al.	2012	MS induces more rapidly progression of DAS than for those without MS [27].
Parhami et al.	1997	In vitro, oxidized lipid products and hypercholesterolemia has induced aortic valve calcification and stenosis [28].
Vongpromek et al.	2015	Hypercholesterolemia induced aortic valve calcinosis and DAS [29].
Parisi et al.	2015	Increased LDL levels activates calcinosis of aortic valve [30].
CHR study	1991	Cholesterol and related lipoproteins are independent risk factors of DAS [31].
MONICA/KORA study	2009	Hypercholesterolemia and active smoking were significantly related to DAS at follow-up [32].
Epic-Norfolk, CCHS, CGPS studies	2014	Elevated Lp(a) levels increase the risk of aortic valve calcinosis [33,34].
MESA study	2016	Lp(a) levels are associated with aortic valve calcinosis [35].
Rajamannan et al.	2016	Lp(a) levels is associated with DAS by genetic variations [36]
CHARGE study	2014	Lp(a) directly induces calcinosis of aortic valve and progression of DAS. Genetic risk score of LDL was significantly associated with calcinosis of aortic valve [37,38].
Thanassoulis et al.	2016	Targeted Lp(a) therapy may become a new opportunity to treat DAS [39].
Chen et al.	2018	2 LPA variants (rs10455872 and rs3798220) was associated with aortic stenosis [40].
Kamath et al.	2008	DAS progresses twice as rapidly in cases of hypercholesterolemia [41].
Kücük et al.	2015	In vitro, HDL is characterized by anti-calcinotic properties. It protects LDL from the oxidation process [42].

DAS—degenerative aortic stenosis.

**Table 2:** Studies assessing the role of lipid-reducing therapy effect in case of DAS.

Study	Data	Results
SALTIRE study (Prospective randomized)	2005	Intensive lipid-lowering therapy does not halt the progression of calcific aortic stenosis or induce its regression for patients with normal cholesterol levels [45].
RAAVE study (Prospective non-randomized)	2007	Statin therapy is useful in case of hyperlipidemia only [44].
SEAS study (Prospective randomized)	2007	Trials have not found any effect of intensive lipid-reducing therapy in case of DAS [46].
Miller et al.	2009/2010	Lipid-reducing therapy stops progression—but does not induce regression—of aortic valve stenosis in hypercholesterolemic mice [13].
ASTRONOMER study (Prospective randomized)	2010	Cholesterol lowering with rosuvastatin did not reduce the progression of DAS in patients with mild to moderate DAS [47].
Greve et al.	2018	The lipid-lowering therapy effect on slowing AS progression increased with higher pretreatment LDL and lower peak aortic jet velocity [49].

DAS—degenerative aortic stenosis.
